# The Regulatory Role of Long Noncoding RNAs in Different Brain Cell Types Involved in Ischemic Stroke

**DOI:** 10.3389/fnmol.2019.00061

**Published:** 2019-03-22

**Authors:** Runsen Chen, Xiangming Xu, Lidan Huang, Wangtao Zhong, Lili Cui

**Affiliations:** ^1^Guangdong Key Laboratory of Age-Related Cardiac and Cerebral Diseases, Affiliated Hospital of Guangdong Medical University, Zhanjiang, China; ^2^Department of Neurology, Affiliated Hospital of Guangdong Medical University, Zhanjiang, China

**Keywords:** long noncoding RNA, ischemic stroke, biomarker, mechanism, neuron, microglia, vascular endothelial cell

## Abstract

Stroke results in high morbidity and high mortality worldwide, with ischemic stroke accounting for 80% to 85%. As effective treatments for ischemic stroke remain limited because of the narrow therapeutic time window, a better understanding of the pathologic mechanism and new therapeutic intervention targets are needed. Due to the development of next-generation sequencing technologies and the genome-wide analysis of eukaryotic transcriptomes, a large amount of evidence to date demonstrates that long noncoding RNAs (lncRNAs) play a vital role in gene regulation and in ischemic stroke. In recent years, many studies have been focused on the clinical significance of lncRNAs in ischemic stroke, and data shows that the pathological processes underlying ischemic stroke are driven by interactions among different brain cell types, including neurons, glial cells, and vascular cells, which actively participate in the mechanisms of tissue injury and repair. In this mini review article, we provide an overview of the characteristics and underlying regulation mechanisms of lncRNAs relevant to different brain cell types during the course of ischemic stroke. Moreover, we reveal the roles of lncRNAs as potential biomarkers and treatment targets in ischemic stroke.

## Introduction

Stroke has high morbidity and high mortality worldwide and is also a major cause of long-term disability in both developing and developed countries. Among all subtypes, ischemic stroke accounts for 80% to 85% of stroke cases (Tobin et al., 2014). Despite efforts to develop treatments using intravenous and intra-arterial therapies, effective treatments for ischemic stroke are still limited. For example, thrombolytic therapy for the treatment of acute ischemic stroke shows restricted efficacy, and only a small percentage of patients can receive this therapy because of the narrow therapeutic time window (Schwamm et al., 2013). Similar to intravenous treatment, mechanical thrombectomy should be initiated as quickly as possible (Powers et al., 2018). Nonetheless, severe treatment complications, such as intracranial hemorrhage in patients treated with an intravenous injection of thrombolytic agents, are associated with high mortality (Seet and Rabinstein, 2012). Overall, it is necessary to clarify the underlying pathological mechanisms of ischemic stroke.

According to sequencing of the human transcriptome, more than 68% is composed of long noncoding RNAs (lncRNAs; Carninci et al., 2005; Iyer et al., 2015), which are long transcripts (>200 bp) similar to mRNAs but lack protein-coding potentials that were once considered to be transcriptional noise (Moskowitz et al., 2010; Derrien et al., 2012). Some scholars subclassify lncRNAs as large intergenic (or intervening) ncRNAs (lincRNAs) and transcribed ultraconserved regions (T-UCRs). LincRNAs, which comprise the majority of lncRNAs, are situated between coding and noncoding genes and do not overlap the exons of other genes (Guttman et al., 2011), with lincRNAs being the least conserved and T-UCRs being the most conserved between mammals and rodents (Bejerano et al., 2004). LncRNAs actively regulate the expression and function of protein-coding genes *via* different mechanisms (Mercer et al., 2009). Although many studies have demonstrated that lncRNAs are involved in diverse diseases such as ischemic stroke, their functions are currently unclear. Nonetheless, a rising number of studies have focused on the clinical significance of lncRNAs in ischemic stroke. In this mini review article, we provide an overview of the expression characteristics of lncRNAs according to different brain cell types and reveal that they might serve as biomarkers and therapeutic intervention targets during the course of ischemic stroke.

## Mechanism of Long Noncoding RNAs in Stroke

**Figure 1:**
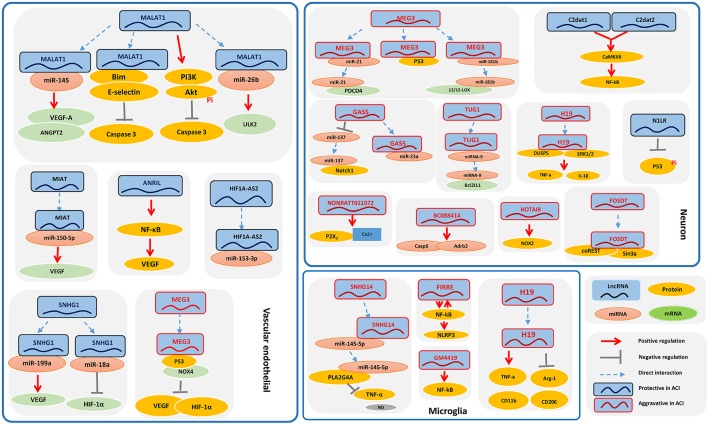
The signaling pathways of long noncoding RNAs (lncRNAs) in ischemic stroke in different types of brain cells. Some of the lncRNAs play a role in the pathological processes underlying ischemic stroke among different brain cell types, mainly including neurons, glial cells, and vascular cells, which actively participate in the mechanisms of tissue injury and repair, which are also the potential therapeutic and preventive targets for IS.

LncRNAs might be involved in the progression of ischemic stroke by participating in such processes as calcium overload, oxidative stress, oxygen deprivation, stress signaling, neurovascular pathophysiology, neuron necrosis, intense inflammatory responses, and synapse function (Mitsios et al., 2006; Deb et al., 2010; Raveendra et al., 2018). Accumulating research indicates that the biological processes underlying stroke are driven by the interaction of neurons, glial cells, vascular cells, and matrix components, which actively participate in the mechanisms of tissue injury and repair (Moskowitz et al., 2010). Here, we introduce relevant lncRNAs in different types of brain cells according to the current literature (Figure 1).

## Long Noncoding RNAs in Neuronal Cells

Despite great advances in our understanding of the mechanisms of neuronal cell death induced by ischemic stroke, clinically effective neuroprotective therapies are limited (Moskowitz et al., 2010), and additional studies focusing on neurons are needed.

C2dat1 and C2dat2, *CaMK2D*-associated lncRNAs, were discovered in oxygen-glucose deprivation/reoxygenation (OGD/R)-induced neuronal cells (Xu et al., 2016), and it has been reported that these lncRNAs are able to promote neuronal survival. Mechanistically, C2dat1 cooperates with C2dat2 to promote expression of CaMKIIδ and upregulation of IKKα/β and further activates the NF-κB signaling pathway, which activates CaMKII kinases to protect neurons from ischemic damage (Xu et al., 2016; Ye et al., 2019). N1LR, an (ischemic/reperfusion) I/R-induced lncRNA, reportedly enhances cell cycle progression and cell proliferation and inhibits apoptosis in mouse neuronal cells *in vitro*. N1LR was found to inhibit phosphorylation of p53 serine 15 independently of its location-associated gene *Nck1* for neuroprotection. Thus, N1LR may serve as a target for therapeutic intervention following ischemic brain injury (Wu et al., 2017). Recently, a lncRNA GM12371 was discovered in hippocampal neurons, and its expression is essential for regulating synapse function, including synaptic communication, synapse density, synapse morphology, and dendritic tree complexity. Mechanistically, it regulates the expression of several genes involved in neuronal development and differentiation, as well as the expression of specific lncRNAs and their cognate mRNA targets (Raveendra et al., 2018). GM12371 may serve as a vital study target in neuronal cell function.

The lncRNA H19 is upregulated in OGD/R-treated cells *in vitro*, and inhibition of H19 protects cells from OGD/R-induced death by suppressing autophagy through the DUSP5–ERK1/2 axis. Based on blood analyses, *H19* gene variation increases the risk of ischemic stroke. Taken together, these results suggest that H19 might be a potential therapeutic target in ischemic stroke (Wang et al., 2017a). BC088414, found in ischemic brains, is correlated with apoptosis-related genes, including *Casp6* and *Adrb2*. Silencing of BC088414 was reported to reduce mRNA levels of *Casp6* and *Adrb2* and apoptosis and to promote cell proliferation *in vitro*. Mechanistically, BC088414 might promote apoptosis through *Adrb2* and *CASP6* and aggravate neuronal cell injury induced by hypoxia–ischemia (Zhao F. Y. et al., 2015, Zhao F. et al., 2015). Consistent with increased NOX2 production, HOTAIR is significantly upregulated in hypoxia-treated mouse neuronal cells, and strong expression of HOTAIR promotes the onset of ischemic infarct by regulating NOX2 expression, which offers clues regarding the molecular mechanisms involved in ischemic stroke (Yang and Lu, 2016). Expression of FosDT (*Fos* Downstream Transcript), which is cogenic with the *Fos* gene, was found to be induced in a transient ischemic rat model, whereas FosDT knockdown significantly ameliorated post-ischemic motor deficits and reduced the infarct volume. FosDT inhibits downstream genes of the REST complex, such as *GluR2*, *NF-κB2*, and* N-methyl-D-aspartate 1*, by forming a complex with coREST and Sin3a. Accordingly, FosDT might be a therapeutic target for minimizing post-stroke brain damage (Mehta et al., 2015). In addition, lncRNA TUG1 is significantly upregulated in OGD/R-induced neurons and acts as a sponge for microRNA-9 to promote apoptosis by upregulating the protein Bcl-2-like 11 (Bcl2l11). These findings show the role of TUG1, possibly providing a new potential therapeutic target in stroke (Chen et al., 2017). Growth arrest-specific 5 (GAS5) is significantly upregulated in OGD-treated neuronal cells, functioning as a competing endogenous RNA (ceRNA) for miR-137 to reduce neuronal survival through inactivation of the Notch1 signaling pathway (Chen et al., 2018). It was reported that intracerebroventricular injection of GAS5 shRNA significantly decreased brain GAS5 expression, inhibiting miR-23a, reducing brain infarct size, and improving recovery of neurological function. These results indicate that GAS5 might promote the progression of ischemic stroke (Zhao et al., 2018). In one study, lncRNA NONRATT021972 was significantly upregulated in OGD/R-induced neuronal cells, and NONRATT021972 siRNA suppressed upregulation of the P2X7 receptor and reduced the P2X7 receptor-mediated neuronal injury induced by OGD (Li et al., 2016). This treatment also prevented pathological changes in cell viability and calcium overload as well as sympathetic neuronal injury following ischemia in a mouse model. Therefore, treatment with NONRATT021972 siRNA might be a potential approach for ischemic stroke (Li et al., 2016).

Several lncRNAs, such as maternally expressed gene 3 (MEG3), influence the pathological course of ischemic stroke through different mechanisms such as decreasing the expression of pro-angiogenic factors, inducing apoptosis, and promoting ischemic damage (Liu et al., 2016; Yan et al., 2016, 2017; Zhan et al., 2017). Below, we provide some examples of the effects of lncRNAs on neurons; their functions of other types of brain cells will be discussed in the following sections. First, MEG3 expression was upregulated in mice following ischemia, and p53 was recruited to the MEG3 complex in ischemic tissues. MEG3 bound directly to the p53 DNA-binding domain, stimulating p53-mediated transactivation and ischemic neuronal death (Yan et al., 2016). Second, MEG3 functioned as a ceRNA and competed with *programmed* *cell death 4* (*PDCD4*) mRNA for direct binding to miR-21, which mediated ischemic neuronal death. Moreover, knockdown of MEG3 protected against ischemic damage and improved overall neurological functions *in vivo* (Yan et al., 2017). In another study, knockdown of MEG3 contributed to the attenuation of apoptosis in OGD-treated mouse neuronal cells. In contrast to MEG3, miR-181b overexpression reduced apoptosis and suppressed the hypoxia-induced increase in 12/15-LOX expression. Together, MEG3 functions as a ceRNA for miR-181b to regulate 12/15-LOX expression in OGD-cultured mouse neuronal cells (Liu et al., 2016). These results might help to develop new strategies for therapeutic interventions in ischemic stroke.

## Long Noncoding RNAs in Vascular Endothelial Cells

Vascular abnormalities might contribute to the pathogenesis of many neurological disorders (Zacchigna et al., 2008). Indeed, angiogenesis serves as an important protective mechanism against ischemic stroke, as it promotes the generation of collateral circulation and consequently increases the blood supply to areas of cerebral infarction (Li L. et al., 2017). Some microarray profiling studies (Dharap et al., 2012, 2013; Vemuganti, 2013; Zhang et al., 2016) have revealed the vital role of lncRNAs in the pathogenesis of ischemic stroke, which may contribute to a better understanding of ischemic stroke and provide novel therapeutic options. Below, we introduce the endothelial-selective lncRNAs studied in animal experiments.

LncRNA ANRIL was significantly increased in rat cerebral infarction, promoting angiogenesis and inflammation processes. Mechanistically, ANRIL overexpression upregulated VEGF and promoted angiogenesis by activating the NF-κB pathway (Zhang B. et al., 2017). Additionally, lncRNA small nucleolar rna host gene 1 (SNHG1) expression was markedly increased in both cerebral microvessels of mice with transient ischemia and OGD-treated brain microvascular endothelial cells (BMECs). Inhibition of this lncRNA resulted in a larger brain infarct size and worse neurological scores in mice and increased caspase 3 activity and apoptosis in cells. SNHG1 was found to function as a ceRNA for miR-18a, regulating the de-repression of its endogenous target HIF-1α and promoting BMEC survival through HIF-1α/VEGF signaling (Zhang et al., 2018). Another study showed that SNHG1 promoted angiogenesis in BMECs after OGD treatment through the targeting of miR-199a (Wang Z. et al., 2018). A protective role in regulating vascular function was reported for MIAT, and its expression was significantly upregulated in endothelial cells in response to hypoxic or oxidative stress. Conversely, knockdown of MIAT led to cerebral microvascular degeneration, progressive neuronal loss, neurodegeneration, and behavioral deficits in a central nervous system (CNS) neurovascular disorder. Mechanistically, MIAT regulates neural and vascular cell function *via* the MIAT/miR-150-5p/VEGF network (Jiang et al., 2016). In another study, MIAT knockdown obviously ameliorated diabetes-induced retinal microvascular dysfunction *in vivo* and inhibited endothelial cell proliferation, migration, and tube formation *in vitro* (Yan et al., 2015). Overall, MIAT might represent a pharmacological target for treating neurovascular-related disorders (Yan et al., 2015; Jiang et al., 2016). HIF1A-AS2 was upregulated in human umbilical vein endothelial cells (HUVECs) in hypoxia, enhancing angiogenesis by acting like a “sponge” for miR-153-3p, which decreased the post-transcriptional silencing of HIF-1α by miR-153-3p. Furthermore, this function of HIF1A-AS2 facilitated activation of HIF-1α/VEGFA/Notch1 cascades to promote HUVEC viability, migration ability, and tube formation (Li L. et al., 2017).

As mentioned above, MEG3 influences the pathological processes of neurons, and the function of MEG3 in vascular endothelial cells is clarified below. MEG3 and NOX4 expression are increased in OGD/R-treated rat BMECs, and inhibition of MEG3 protects BMECs against apoptosis by reducing NOX4 and *p53* expression and further enhancing that of pro-angiogenic factors (HIF-1ocesses of nand decreasing intracellular reactive oxygen species generation. Regarding its mechanism, MEG3 promotes apoptosis in BMECs and reduces angiogenesis *via* regulation of the p53/NOX4 axis (Zhan et al., 2017).

Metastasis-associated lung adenocarcinoma transcript one (Malat1) is a highly upregulated I/R- or OGD/R-responsive endothelial lncRNA and plays a protective role in BMECs (Li Z. et al., 2017). Malat1 might participate in stroke development through multiple mechanisms, including promoting angiogenesis, BMEC autophagy, and survival; binding to specific factors; regulating proliferative and migratory activities; and activating signaling pathways. First, Malat1 directly regulates miR-145, which was revealed to have an inhibitory function on angiogenesis by directly targeting VEGF-A and ANGPT2 (Ren et al., 2019). Second, Malat1 acts as an endogenous sponge to downregulate miR-26b expression by binding directly to miR-26b. Malat1 reverses the inhibitory effect of miR-26b on BMEC autophagy and survival, which is involved in promoting the expression of the miR-26b target ULK2 (Li Z. et al., 2017). Third, Malat1 binds to Bim and E-selectin, a proapoptotic factor and proinflammatory cytokine, respectively, decreasing OGD-induced cell death and caspase 3 activity in BMECs (Zhang X. et al., 2017). Moreover, silencing of Malat1 promotes a migratory rather than a proliferative endothelial cell phenotype *in vitro*, and its genetic deletion or pharmacological inhibition reduces vascular growth *in vivo* (Michalik et al., 2014). Lentiviral overexpression of Malat1 increases phosphatidylinositol 3-kinase (PI3K) activity and activation of protein kinase B phosphorylation and decreases apoptosis and caspase 3 activity, which suggests that Malat1 might protect human BMECs from OGD/R-induced apoptosis *via* a PI3K-dependent mechanism (Xin and Jiang, 2017; Wang G. et al., 2018). These findings suggest that MALAT1 is a potential therapeutic target for ischemic stroke.

## Long Noncoding RNAs in Microglial Cells

Cells other than neurons may also be involved in the pathogenesis of ischemia, and a functional “neurovascular unit” comprises neuronal, glial, and vascular elements (Zacchigna et al., 2008; del Zoppo, 2009). Based on the literature, researchers prefer to use microglia as study subjects compared to other types of glial cells. Therefore, we discuss the role of microglial cells in ischemic stroke in this section.

H19 expression is increased in the plasma, white blood cells, and the brain of middle cerebral artery occlusion (MCAO)-treated mice. Intracerebroventricular injection of H19 siRNA reduces infarct volume and brain edema, decreases tumor necrosis factor-α and interleukin-1β levels in brain tissue and plasma, and increases plasma interleukin-10 levels 24 h post-stroke. Mechanistically, H19 promotes neuroinflammation by driving HDAC1-dependent M1 microglial polarization, suggesting a potential novel H19-based diagnosis and therapy for ischemic stroke (Wang et al., 2017b). Gm4419 is abnormally upregulated in OGD/R-treated microglial cells and causes neuroinflammatory damage. Knockdown of Gm4419 causes inhibition of NF-κB, showing that downregulation of Gm4419 has a protective role against OGD/R injury, and Gm4419 is found to function as a crucial mediator in NF-κB pathway activation (Wen et al., 2017). Functional intergenic repeating RNA element (FIRRE) is found to be upregulated and associated with injury in OGD/R-treated microglial cells, forming a positive feedback loop with NF-κB to promote transcription of the NLRP3 inflammasome, thereby contributing to OGD/R injury of cerebral microglial cells (Zang et al., 2018). SNHG14, also called the antisense of ubiquitin protein ligase E3A (UBE3A-ATS; Stanurova et al., 2016), is significantly upregulated in OGD-treated BV-2 microglia cells, which leads to apoptosis and expression of proinflammatory factors such as TNF-1α and nitric oxide (NO). SNHG14 acts as a sponge to inactivate miR-145-5p, thereby enhancing the expression of PLA2G4A, which results in the activation of microglia cells in the cerebral infarction (Qi et al., 2017).

## Potential Biomarkers

The potential of lncRNAs to regulate physiological and pathological processes highlights their potential therapeutic importance. In theory, each lncRNA associated with ischemic stroke has the potential to serve as a therapeutic target or prognostic marker. However, whether a specific lncRNA can be applied for clinical use is affected by complicating factors. Below, we mainly focus on the expression characteristics of relevant lncRNAs studied in patients.

## Time Dependency of Long Noncoding Rnas

Expression of stroke-relevant lncRNAs is dependent on the reperfusion time. In one study, 259 lncRNA isoforms at 6 h, 378 isoforms at 12 h, and 217 isoforms at 24 h following reperfusion were differentially expressed compared to sham controls, and a reperfusion time-specific analyses revealed that lncRNAs reached peak expression levels at 6 h following reperfusion (Liu et al., 2016). Transient focal ischemia induces widespread changes in lncRNA expression, and the characteristics of distinct reperfusion time-point-dependent expressions reveal that lncRNAs might participate in the progression of ischemic pathophysiology (Bhattarai et al., 2017).

Time-dependent expression may result from different half-lives (*t*_1/2_). For example, analysis of the *t*_1/2_ of lncRNAs and mRNAs in the mouse Neuro-2a cell line showed that only a minority of lncRNAs are unstable. LncRNA *t*_1/2_ vary over a wide range, comparable to that of mRNAs, though on average less, which is suggestive of complex metabolism and widespread functionality. In addition, combining *t*_1/2_ with comprehensive lncRNA annotations identified hundreds of unstable (*t*_1/2_ < 2 h) cis-antisense, intronic and intergenic lncRNAs, as well as lncRNAs showing extreme stability (*t*_1/2_ > 16 h; Clark et al., 2012). Moreover, some studies have reported that lincRNAs with short half-lives (*t*_1/2_ < 4 h) are well-known regulatory lincRNAs and that several ncRNAs with long half-lives (*t*_1/2_ ≥ 4 h) are involved in housekeeping functions (Tani et al., 2012), similar to mRNAs. Because the median lncRNA *t*_1/2_ is 3.5 h (Clark et al., 2012), an unstable lincRNA is defined as that with a *t*_1/2_ less than 4 h and a stable lincRNA as that with a *t*_1/2_ greater than or equal to 4 h. Some relatively stable lincRNAs with long *t*_1/2_ may serve “housekeeping” roles (Wang et al., 2014). The strategy of monitoring RNA *t*_1/2_ will provide a powerful tool for investigating hitherto functionally uncharacterized regulatory RNAs (Tani et al., 2012).

## Potential Markers

The expression levels of lncRNAs change significantly in the brain and blood after ischemic stroke, suggesting that lncRNAs are potential biomarkers for this event (Dykstra-Aiello et al., 2016), and several studies have identified the differential expression of lncRNA in mouse models (Dharap et al., 2012; Liu et al., 2018). Despite recent progress in clinical research on lncRNA markers for stroke, application of lncRNAs as biomarkers in the clinic has not yet been realized, because detailed mechanistic studies are lacking for many of them. Below, we discuss lncRNAs that have been evaluated in patients (Table 1).

**Table 1:** LncRNAs from clinical research in IS patients.

LncRNA	Level	Case number	Tissue
linc-DHFRL1–4 (Deng et al., 2018)	Up	IS patients (*n* = 206), TIA patients (*n* = 55)	PBMC
		Controls (*n* = 179)
SNHG15 (Deng et al., 2018)	Up	IS patients (*n* = 206), TIA patients (*n* = 55)	PBMC
		Controls (*n* = 179)
linc-FAM98A-3 (Deng et al., 2018)	Up	IS patients (*n* = 206), TIA patients (*n* = 55)	PBMC
		Controls (*n* = 179)
lncRNA-RMST (Hou and Cheng, 2018)	Up	IS patients (*n* = 10)	Plasma
		Controls (*n* = 10)
MIAT (Zhu et al., 2018)	Up	IS patients (*n* = 189)	Peripheral blood leukocytes
		Controls (*n* = 189)
H19 (Wang et al., 2017b)	Up	IS patients (*n* = 36)	Plasma white blood cells
		Controls (*n* = 25)
BDNF-AS (Zhong et al., 2017)	Up	IS patients (*n* = 20)	Brain tissue
		Control (*n* = 5)

Some lncRNAs have been reported to have the potential to act as biomarkers, such as lncRNAs RMST and SNHG15 and lincRNAs FAM98A-3 and DHFRL1–4 (Deng et al., 2018; Hou and Cheng, 2018). To investigate the expression profile of lncRNAs in peripheral blood mononuclear cells (PBMCs) from acute ischemic stroke patients and to explore their utility as biomarkers, a clinical study involving 206 ischemic stroke patients, 179 healthy controls (HCs), and 55 patients with transient ischemic attack (TIA) was conducted. According to the results, lncRNAs (SNHG15 and linc-FAM98A-3) were significantly upregulated in ischemic stroke patients compared to HCs and TIA patients. Furthermore, longitudinal analysis of lncRNA expression up to 90 days after ischemic stroke showed that lincRNA FAM98A-3 had normalized to control levels by day 7, whereas SNHG15 levels remained high, indicating the ability of lncRNAs to monitor ischemic stroke dynamics and serve as a potential novel diagnostic tool (Deng et al., 2018). RMST, the expression of which was significantly upregulated in OGD-treated neurons, the MCAO-induced injured brain, and the plasma of patients with ischemic stroke, was found to be involved in the pathogenesis of ischemic brain injury. In addition, RMST silencing protected against ischemic injury; thus, RMST inhibition has potential for treating ischemic stroke (Hou and Cheng, 2018). In another study, MIAT expression was significantly upregulated in peripheral blood leukocytes of 189 ischemic stroke patients compared to controls, and this increase was markedly correlated with National Institutes of Health Stroke Scale (NIHSS) scores, mRS scores, high-sensitivity C-reactive protein, and infarct volume. Additionally, ischemic stroke patients with increased MIAT expression had a relatively poor prognosis compared to a group with low MIAT expression (Zhu et al., 2018). Similarly, BDNF-AS (antisense RNA brain-derived neurotrophic factor) was significantly upregulated in brain tissues from 20 ischemic stroke patients, and BDNF-AS siRNA inhibited hypoxia/reoxygenation (HR)-induced apoptosis and ameliorated H/R-induced reduction of mitochondrial membrane potential by activating the BDNF–TrkB–PI3K/Akt pathway following H/R-induced neurotoxicity (Zhong et al., 2017). Circulating H19 levels, which were significantly higher in 36 stroke patients compared with 25 HCs, showed a positive correlation with NIHSS scores and tumor necrosis factor-α levels. Furthermore, H19 knockdown attenuated brain tissue loss and neurological deficits 14 days post-stroke by promoting neuroinflammation to drive microglial polarization, suggesting a potential diagnosis and therapy for ischemic stroke (Wang et al., 2017b).

## Conclusion and Perspective

A growing number of studies have illustrated that lncRNAs might play crucial roles in the regulation of pathophysiological processes in ischemic stroke and might be potential therapeutic targets. However, clinical research on lncRNAs is still in its infancy, and many challenges remain. First, many lncRNAs are not conserved at the sequence level, and there is no systematic and scientific catalog of lncRNAs to date. Many classic methods that have been applied to coding genes have proven less useful for deciphering the elaborate populations of transcripts generated by pervasive lncRNA transcription. Second, the traditional lncRNA definition may be changed in the future: lncRNAs are typically defined as ncRNAs that are longer than 200 nucleotides and do not encode proteins; however, as many lncRNAs have been revealed to encode short peptides in mammalian tissues and with other new functions, this typical definition might need to be redefined (Wilhelm et al., 2014; Anderson et al., 2015). Third, expression of lncRNAs is time point dependent, and half-lives vary. Thus, how to unify time points to study these lncRNAs in acute ischemic stroke patients is important and challenging. In addition, compared to protein-coding genes and miRNAs, lncRNAs exhibit higher tissue specificity but are less conserved among species (Xu et al., 2017).

The number of samples that can be obtained from patients is still relatively small. Furthermore, because of the subjects used and the tissues studied and because the types of specimens collected are not unifying, the results and conclusions from these studies require further confirmation before clinical application. Moreover, many important discoveries in this field are based on animal studies, and additional work focusing on patients and on clinical applications should be conducted. Research incorporating new scientific methods, larger clinical samples, and standardized time points for analyzing samples are encouraged. Overall, developing a highly sensitive biomarker for ischemic stroke is challenging but interesting.

## Author Contributions

LC formulated the original idea and reviewed and approved the manuscript. WZ contributed to the design of the review. RC wrote and reviewed the manuscript and designed the table and figures. XX and LH contributed to the design of the figures.

## Conflict of Interest Statement

The authors declare that the research was conducted in the absence of any commercial or financial relationships that could be construed as a potential conflict of interest.
